# Green Synthesized Copper Oxide Nanoparticles Ameliorate Defence and Antioxidant Enzymes in *Lens culinaris*

**DOI:** 10.3390/nano10020312

**Published:** 2020-02-12

**Authors:** Joy Sarkar, Nilanjan Chakraborty, Arindam Chatterjee, Avisek Bhattacharjee, Disha Dasgupta, Krishnendu Acharya

**Affiliations:** 1 Department of Botany, Dinabandhu Andrews College, Garia, Kolkata 700084, India; jsarkar80@gmail.com; 2 Department of Botany, Scottish Church College, Kolkata 700006, India; nilanjanchak85@gmail.com (N.C.); avisek0007@gmail.com (A.B.); dishadasgupta5@gmail.com (D.D.); 3 Department of Botany, University of Kalyani, Kalyani, Nadia 741235, India; arindamchatterjee206@gmail.com; 4 Molecular and Applied Mycology and Plant Pathology Laboratory, Centre of Advanced Study, Department of Botany, University of Calcutta, Kolkata 700019, India

**Keywords:** *Adiantum lunulatum*, antioxidative enzymes, copper oxide nanoparticles, defence enzymes, reactive oxygen species, nitric oxide, transmission electron microscope

## Abstract

Biosynthesis of copper oxide nanoparticles (CuONPs) in a cost-effective and eco-friendly way has gained its importance. CuONPs has been prepared from copper sulfate by using *Adiantum lunulatum* whole plant extract. CuONPs have been characterized by X-ray diffraction, Fourier transform infrared spectroscopic, transmission electron microscope, etc. Mono-disperse, spherical, pure, and highly stable CuONPs have formed with an average diameter of 6.5 ± 1.5 nm. Biosynthesized CuONPs at different concentrations were applied to seeds of *Lens culinaris*. Physiological characteristics were investigated in the germinated seeds. Roots obtained from the seeds treated with 0.025 mgmL^−1^ concentration of CuONPs showed highest activity of different defence enzymes and total phenolics. However, at higher concentration it becomes close to control. It showed gradual increase of antioxidative enzymes, in accordance with the increasing dose of CuONPs. Likewise, lipid peroxidation and proline content gradually increased with the increasing concentration. Reactive oxygen species and nitric oxide generation was also altered due to CuONPs treatment indicating stress signal transduction. Finally, this study provides a new approach of the production of valuable CuONPs, is a unique, economical, and handy tool for large scale saleable production which can also be used as a potent plant defence booster instead of other commercial uses.

## 1. Introduction

Copper (Cu) is one of the indispensable microelements obligatory for the growth and development of plant. It can be present as Cu^2+^ and Cu^+^ under natural conditions. Optimum concentration is regularly involved in the plants, ranging from 10^−14^ to 10^−16^ M. In addition to many of its important functions such as cell wall metabolism and protein regulation, it also acts as secondary signaling molecule in plant cells. It takes part in the mitochondrial respiration, photosynthetic electron transport, iron mobilization, hormone signaling, oxidative stress response, and also acts as cofactor for many enzymes [1,2]. However, a higher dose of Cu leads to oxidative stress generation, growth inhibition, cellular malfunctioning, and photosynthesis retardation [2,3,4].

Though, nanotechnology is considered to be the next industrial revolution, the search is still ongoing for better nontoxic, hazard free, and eco-friendly approach for synthesis of nanomaterials [5]. In comparison to chemically synthesized nanoparticles, green synthesized nanoparticles are more effective and eco-friendly [6]. Until now various biological organisms are reported to have their potentiality for the production of metallic nanoparticles. However, the rate of metal nanoparticle synthesis with the help of plant extract is stable [7], much faster [8,9], and extremely mono-dispersive [10] in respect to other biological methods. Moreover, in the case of copper, different organisms suchas microbes [11,12], algae [13], fungi [14,15], and angiosperm plant extracts [16,17], are utilized for the nanoparticles production.

Among other cryptograms, algae [7] and bryophytes [18] are quite popular to fabricate metal nanoparticles. However, potentiality of different pteridophytes (fern and fern allied species) for the production of nanoparticles is less surveyed. Until now only few species such as *Pteris tripartite* [19], *Adiantum capillus-veneris* [20], *A. caudatum* [21], *A. philippense* [22,23], *Asplenium scolopendrium* [24], *Actinopteris radiata* [25], *Azolla microphylla* [26], etc. have been used.

Although previously there are some reports where some ferns are considered to be poisonous and carcinogenic for the animals [27]. However, our choice of interest, *Adiantum lunulatum* Burm. f. is popular due to its antimicrobial [28], antioxidant [29], and other medicinal properties. The plant extract may act as an anti-hyperglycemic [30] and also have some curative role against influenza and tuberculosis [31]. The plant is a rich source of various terpenoids, carbohydrates, phenols, and flavonoids [29,32,33].

On the other hand, despite other inorganic applications, nanomaterials are utilized as an antimicrobial agent and defence booster in the field of agriculture. Recently, we have reported the effect of chitosan nanoparticles as an inducer of innate immunity in tea [34]. Recent research on nanoparticles in a number of crops such as corn, wheat, ryegrass, alfalfa, soybean, tomato, radish, lettuce, spinach, onion, pumpkin, bitter melon, and cucumber have provided evidence of enhanced seedling growth, germination, photosynthetic activity, nitrogen metabolism, protein level, mRNA expression, and changes in gene expression indicating their potential use for crop improvement.

In this context, here, an attempt has been made to synthesize copper oxide nanoparticles in an eco-friendly, greener route by using the important fern *Adiantum lunulatum*. The potentiality of this CuONPs in the induction of defence and generation of stress has also been checked in a model plant *Lens culinaris*.

## 2. Materials and Methods

### 2.1. Collection and Identification of Plant Material

The fresh plant was collected from the Rajpur-Sonarpur Municipality area (22.4491° N, 88.3915° E) of the district South 24 Parganas, West Bengal, India. The respective plant specimen was self-identified and binomially jointly by Pteridology and Paleobotany Lab, Department of Botany, University of Kalyani, Kalyani, Nadia, West Bengal, India, Pin-741235 and Department of Botany, Dinabandhu Andrews College, Garia, Kolkata, West Bengal, India, Pin-700084. Voucher specimens were prepared from the collected specimens and further deposited both at the Herbarium of the Botany Department, University of Kalyani, as well as Herbarium of the Botany Department, Dinabandhu Andrews College, Kolkata.

### 2.2. Preparation of Plant Extract

The whole plant was rinsed with tap water and distilled water, respectively. The superficial water was soaked from the plant surface. Fiveg of the desiccated plant material was homogenized in mortar and pestle into a paste. After that, 100 mL of distilled water was mixed to that paste [22]. Filtration of the crude solution was done thrice by Whatman filter paper no.1. Finally, the filtrate was collected all together for future reference.

### 2.3. Green Synthesis of Copper Oxide Nanoparticles

For the production of copper oxide nanoparticles, the plant extract was used to reduce copper sulfate (CuSO_4_-1 mM). The substrate was mixed with the plant extract (ratio: 1:5, *v*/*v*) and stirred constantly for at least 1 h [35,36]. Simultaneously, a positive control set (only the plant extract of *A. lunulatum*) and a negative control set (only the copper sulfate solutions without plant extract) was also maintained at a similar experimental condition. The reaction mixtures were regularly monitored and the color change was recorded properly. The reactions were performed under normal room temperature (37 °C) at pH 9 (optimum).

The whole preparation was centrifuged at 12,000× *g* for 15 min and the pellet containing nanoparticles were washed (three times) with deionized water. The purified CuONPs were resuspended in deionized water and ultra-sonicated by a Piezo-u-sonic ultrasonic cleaner (Pus-60w) and kept at normal room temperature (37 °C) [35].

### 2.4. Characterization of Copper Nanoparticles

#### 2.4.1. UV–Visible Spectroscopy of Synthesized Nanoparticles

Plant extract mediated biosynthesized nanoparticles had been observed under a Hitachi 330 spectrophotometer with plasmon peaks at different regions of the spectral range 200–900 nm, which corresponded to different signature marks for the production of different nanoparticles, respectively [37]. Deionized water was used as reference.

#### 2.4.2. Particle Size Measurement by Dynamic Light Scattering (DLS) Experiment

By laser diffractometry, particle size was measured using a nano-size particle analyzer (Zen 1600 Malvern USA) in the range between 0.6 and 6.0 μm, under such conditions such as having particle refractive index 1.590, particle absorption coefficient 0.01, water refractive index 1.33, viscosity—cP, Temperature-25 °C and general calculation model for irregular particles. About 10−15 measurement cycles of 10 s each were taken and the data obtained were averaged by the respective instrument preloaded software (DTS, version 5.00 from Malvern) [38].

#### 2.4.3. Transmission Electron Microscopic (TEM) Observation of Nanoparticles

Morphological and topographical characterization of the nanoparticles had been well established by the electron microscopic studies [39]. On a carbon coated copper grid thin films of the synthesized nanoparticles were prepared (30 × 30 μm mesh size) and a drop of the nanoparticle suspension was spotted on the grid. Excess solution was removed by using blotting paper. It was allowed to dry under a mercury lamp for 5 min. The micrographs were obtained by Tecnai G2 spirit Biotwin (FP 5018/40) instrument, operating at around 80 kV accelerating voltage [14].

#### 2.4.4. Zeta Potential Measurement

Using Beckman Coulter DelsaTM Nano Particle Analyzer (USA) zeta potential (Charge distribution) of the nanoparticles was investigated by illuminating the solution with He–Ne laser (658 nm) in a sample cell. Using phase analysis light scattering mode measurements were taken with an Ag electrode [40].

#### 2.4.5. Investigation of Elemental Compositions of the Nanoparticles by Energy Dispersive Spectroscopy (EDX)

A small amount of the sample was taken in glass slide creating a thin layer of sample. Extra solution was rinsed using a blotting paper and then the sample was allowed to dry overnight [41]. This analysis of the samples was carried out by using the Hitachi S 3400N instrument. The spectra were recorded.

#### 2.4.6. XRD Measurement

Crystallinity of forged CuONP was confirmed and determined by XRD analysis. The XRD sample was all set on a microscopic glass slide by depositing the centrifuged sample and thereafter dried at 45 °C in a vacuum drying oven overnight. The vacuum dried CuONPs were then used for powder X-ray diffraction analysis.

The diffractogram was documented from PANalytical, XPERTPRO diffractometer using Cuk (Cu Kα radiation, λ 1.54443) as X-ray source running at 45 kV and 30 mA [42]. The diffracted intensities were noted from 35 to 99° 2θ angles [43].

#### 2.4.7. Fourier Transform Infrared Spectroscopic (FTIR) Analysis

The vacuum dried inorganic metal nanoparticles of culture filtrate were mixed up separately with potassium bromide (KBr), alkali halide at a ratio of 1:100 (*w*/*w*). The two materials were ground to a fine powder in a mortar and pestle separately. Then, the mixture was poured into a pellet press consisting of two pistons in a smooth cylindrical chamber. Pressure of up to 25,000 psi was then applied for different amounts of time in a vacuum. After that the pistons were removed and the clear pellet was placed in a holder of the spectrophotometer. Since the KBr did not absorb infrared radiation in the region 4000 and 400 cm^−1^ a complete spectrum of the solid was obtained [44]. The spectra were recorded with a Shimadzu 8400SFourier transform infrared spectrophotometer using a diffuse reflectance accessory. The scanning data were obtained from the average of 50 scans in the range between 4000 and 400 cm^−1^ [45,46].

### 2.5. Effects of CuONP on Seedling Germination and Seedling Development

*Lens culinaris* Medik was selected as a model plant system to investigate the effects of CuONPs. Seeds of *Lens culinaris* were washed with tap water to remove dirt from seed coats. Then, those seeds were soaked in separate petri-dishes containing water (Control) and three different concentrations of CuONPs (viz. 0.01, 0.025, and 0.05 mgmL^−1^, and kept in the dark for 72 h at 30 ± 2 °C. All the dilutions of CuONPs were prepared from the 1 mgmL^−1^ stock. For each petridish 50 seeds were incubated. After the incubation of three days the seedlings root length and germination percentage was calculated of all the samples [40].

Vigor index of each experimental setup was calculated using the following formula:

Vigor index (VI) = Germination percent (%) × Seedling growth (mm).

### 2.6. Determination of Water Content (WC) and Relative Water Content (RWC)

After four days of germination, fresh weights (FW) of roots from different sets were taken. Then, the roots were immersed in water overnight for turgid weight (TW). It was then ovendried at 100 °C to constant weight and reweighed for the dry weight (DW).

WC was estimated on the FW basis. RWC was determined using the method of [47] and calculated using the following formula:

RWC = [(FW − DW)/(TW − DW)] ×100

Roots of the samples were taken for biochemical and molecular determinations as described below.

### 2.7. Enzyme Extraction and Assays

Fresh root samples (500 mg) were homogenized with 2 mL of 0.1 M sodium phosphate buffer (pH 7.0) containing 0.1% of polyvinyl pyrrolidone (PVP) and 20 µL of 0.05 mM phenyl methane sulfonyl fluoride (PMSF). The extract was centrifuged at 10,000× *g* for 15 min at 4 °C and the supernatant was used to assay the enzyme.

#### 2.7.1. Peroxidase Assay (PO)

The peroxidase (PO) activity was determined by the method of Hemada and klein [48] with few changes. The reaction mixture was prepared with 2.99 mL of substrate (adding 5 mL of 1% guaiacol, 5 mL of 0.3% H_2_ O_2_ mixing, and 50 mL of 0.05 M sodium phosphate buffer) and 0.01 mL of enzyme extract, and the absorption change was measured at 470 nm in a UV–Vis spectrophotometer (Intech, India) for 0–2 min at 30 s interval. The peroxidase activity was determined by the increase in absorbance and expressed as µmolmin^−1^mg^−1^ protein.

#### 2.7.2. Polyphenol Oxidase Assay (PPO)

The polyphenol oxidase (PPO) activity was determined by the method of Kumar and Khan [49] with slight modifications. The reaction mixture was prepared by adding 2 mL of 0.1 M sodium phosphate buffer (pH 6.5), 1 mL of 0.1 M catechol, 0.5 mL of crude enzyme extract and incubated for 10 minat room temperature, and the reaction was ceased by adding 1 mL of 2.5 N H_2_SO_4_. The absorption was measured at 495 nm against a blank set. The PPO activity was taken in U min^−1^mg^−1^ protein. (U = change in 0.1 absorbance min^−1^mg^−1^ tissue).

#### 2.7.3. Phenylalanine Ammonia Lyase (PAL)

Following the same tissue homogenizing procedure for phenylalanine ammonia lyase (PAL) assay a sodium borate buffer (pH 8.7) with PVP and PMSF was used. The method of Dickerson et al. [50] was used for PAL activity to determine the rate of conversion of L-phenylalanine to Transcinnamic acid at 290 nm. The reaction mixture was prepared by adding substrate (1.3 mL of 0.1 M borate buffer, pH 8.7 and 0.5 mL of 12 mM L-phenyl alanine) with 0.2 mL of enzyme and incubated for 30 min at room temperature. The amount of Transcinnamic acid synthesized was measured at 290 nm. Enzyme activity was expressed as nmol of transcinnamic acid min^−1^g^−1^ protein.

#### 2.7.4. β-1,3 glucanase Assay

β-1,3 glucanase activity was assayed using the laminarin dinitrosalicylate method of Pan et al. [51] with minor changes. Root tissues were homogenized in 2 mL of 0.05 M sodium acetate buffer and centrifuged at 10,000× *g* for 15 min at 4 °C. Fifty µL of enzyme and an equal amount of laminarin (1%) was mixed and incubated for 30 min in a 2 mL microcentrifuge tube and at the end of 30 min 0.3 mL of dinitrosalicylic acid reagent was added and boiled for 5 min in water bath. Blank set was prepared in a similar way without mixing of any enzyme in the reaction mixture. Finally, the volume of all the heated microcentrifuge tubes was made to 2 mL by adding distilled water. The microcentrifuge tubes were inverted to mix well and absorption was measured at 520 nm. The enzyme activity was expressed as μmol of glucose released min^−1^g^−1^ protein.

#### 2.7.5. Ascorbate Peroxidase Assay (APX)

Ascorbate peroxidase (APX) activity was determined by following the method of Nakano and Asada [52]. The reaction mixture was prepared by adding 2.9 mL of substrate (containing 50 mM potassium phosphate buffer with pH 7.0, 0.5 mM ascorbic acid, 0.2 mM EDTA, and 2% H_2_O_2_), and 0.1 mL of enzyme extract in a final volume of 3 mL.A decrease in absorbance at 290 nm for 1 min was recorded and using extinction coefficient (ϵ = 2.8 mM^−1^APX was defined as 1 mmolmL^−1^min^−1^cm^−1^) the amount of oxidized ascorbate was calculated as µmolmin^−1^g^−1^ protein.

#### 2.7.6. Catalase Assay (CAT)

Catalase (CAT) assay was determined spectrophotometrically using the method of Cakmak and Horst [53]. The reaction mixture contains 50 µL of H_2_O_2_ (0.3%) with 0.1 mL of enzyme extract and the final volume was made up to 3 mL by adding 50 mM phosphate buffer (pH 7.0). The decrease in absorbance was taken for 0–2 min at 240 nm. The CAT activity was expressed as nmol min^−1^g^−1^ of protein using Molar extinction coefficient, ϵ = 39,400 mM^−1^cm^−1^.

#### 2.7.7. Superoxide Dismutase Assay (SOD)

Activity of superoxide dismutase (SOD) was determined by measuring its ability to inhibit photochemical reduction of nitro blue tetrazolium (NBT) [54]. The reaction mixture contains 0.7 mL of 0.2 M sodium phosphate buffer (pH 7.8), 390 µL of methionine, 300 µL of EDTA (1 mM), 1.2 mL of water, 100 µL of crude enzyme extract, 250 µL of nitroblue tetrazolium (NBT), and 6 µL of riboflavin. Blank was prepared without any enzyme. Two sets were taken and one was incubated in bright light for 10 min and other one was incubated in the dark^.^ After incubation readings were taken at 560 nm, enzyme activity was expressed as percentage of color inhibition mg protein g^−1^.

### 2.8. Estimation of Total Protein Content

Total protein content of the sample was determined using the method of Lowry et al. [55]. The reaction mixture contains 100 µL of extract and volume was made to 1 mL by adding distilled water. Then, 5 mL of Lowry’s reagent was added and incubated for 15 min at room temperature. After this 500 µL of Folinciocalteu reagent was added and incubated in the dark for 30 min, blank sets were taken similarly as absorption was measured at 680 nm. Using the standard curve of BSA solution the amount of protein was calculated and estimated protein was expressed as mg protein g^−1^ of the fresh root sample.

### 2.9. Estimation of Total Phenol Content

Total phenol content was estimated using the method of Zieslin and Benzaken [56] with minor changes. Fresh tissues were homogenized in 2 mL of 80% methanol and maintained for 15 min in water bath at 65 °C, then centrifuged at 10,000× *g* for 10 min at room temperature. The supernatant was used to estimate phenol. The reaction mixture contains 1 mL of extract, 5 mL of distilled water, and 250 µL of 1N Folinciocalteu reagent was added and incubated for 30 min at room temperature. Phenol content was measured spectrophotometrically at 725 nm using gallic acid as standard. The amount of total phenol content was expressed as μg gallic acid produced g^−1^ tissue.

### 2.10. Estimation of Flavonoid Content

The amount of total flavonoid content of the root material was determined using the method of Chang et al. [57]. Fresh tissues were homogenized in 2 mL of 80% ethanol and maintained for 30 min in the dark, then it was centrifuged at 10,000× *g* for 5 min at room temperature. The reaction mixture was prepared with 1 mL of crude extract mixed with 4.3 mL of 80% aqueous ethanol, 0.1 mL of 10% aluminium nitrate, and 0.1 mL of 1 M aqueous sodium acetate and kept in the dark for 30 min. The absorption of the coloured sample was measured at 415 nm against blank sample in a spectrophotometer. The amount of total flavonoid was expressed as µg quercetin g^−1^ fresh tissue.

### 2.11. Determination of Lipid Peroxidation Rate

Using the method of Cakmak and Horst [53], the rate of lipid peroxidation was determined by estimating 2-thiobarbituric acid reactive substances (TBARS) with some modifications. Root samples were grounded in 5 mL of 0.1% (*W*/*V*) trichloroacetic acid (TCA) at 4 °C and centrifuged at 12,000 rpm for 5 min. An aliquot of 1 mL from the supernatant was mixed with 4 mL of 0.5% (*W*/*V*) thiobarbituric acid (TBA) in 20% (*W*/*V*) TCA. Blank set was prepared using distilled water instead of extract. All the reaction mixtures were heated at 90 °C for 30 min. The reactions were stopped in an ice bath and the mixtures were centrifuged at 10,000× *g* for 5 min and the absorbance was taken at 532 nm on a spectrophotometer and corrected for nonspecific turbidity by subtracting the absorbance at 600 nm. The formula was applied to calculate malondialdehyde content using its absorbent coefficient (ϵ) and using the following formula nmolmalondialdehyde g^−1^ fresh mass was calculated.

MDA (nmol g^−1^ FM) = [(A532 − A600) × V × 1000/ϵ] × W

Here, ϵ is the specific extinction coefficient (=155 mMcm^−1^), V is the volume of crushing medium, W is the fresh weight of sample, and A532, A600 are the absorbance at 532 and 600 nm, respectively.

### 2.12. Estimation of Total Proline Content

Using the protocol of Bates et al. [58], the free proline content of samples was determined. Three hundred and fiftymg of root tissues was grounded in 3.5 mL of 3% sulphosalicylic acid in chilled mortar pestle and centrifuged at 11,000× *g* for 15 min at 4 °C. The reaction mixture was prepared with 1 mL of crude extract, 1 mL of 0.5% ninhydrin reagent, and 1 mL of glacial acetic acid. The mixture was boiled for 30 min in a water bath. After cooling it down, 3 mL of toluene was added and shaken. The mixture and upper layer of toluene was collected using a separating funnel. The absorption of the sample was taken at 520 nm against toluene. The amount of proline was determined using standard curve and expressed as µg of proline g^−1^ of tissue.

### 2.13. Estimation of Nitric Oxide (NO)

Real time NO production was visualized using a DAF-2DA membrane permeable fluorochrome dye [59]. Thin sections of roots were taken in loading buffer and 50 µL of 10 mM KCl and 50 µL of 10 mM TrisHCl (pH 7.2) with final concentration of DAF-2DA 10 mM were added and incubated in the dark for 20 min. Fluorescence was observed and high resolution images were taken using Floid Cell Imaging station microscope by Life technologies. Green fluorescence indicates the production of NO.

### 2.14. Measurement of Reactive Oxygen Species (ROS)

ROS generation was monitored according to the method of Gupta et al. [60]. To measure ROS, thin transverse sections of treated roots were immersed in the 1 mL of detection buffer DB (2.5 mM HEPES, pH 7.4) containing 10 µM DCF-2DA fluorescent dye (Invitrogen, Carlsbad, CA, USA), and it was kept for 10 min in dark incubation. The high-resolution images were checked using Floid Cell Imaging station microscope by Life technologies.

### 2.15. In Vivo Detection of H_2_O_2_

In vivo detection of H_2_O_2_ of root samples was carried out using DAB by following the method of Thordal-Christensen et al. [61]. After one week of germination the roots were excised and immersed in a solution containing 1 mg/mL diaminobenzidine (DAB) solution (pH 3.8) and incubated for 8 h in the dark. After that sectioning of roots were performed and the section is immersed in 3:1 (*V*/*V*) ethanol and glacial acetic acid mixture for bleaching. After that it was cleaned with water and for 24 h the sections were dipped in lactoglycerol (1:1:1, lactic acid: glycerol:water *V*/*V*). High-resolution images were taken using Floid Cell Imaging station microscope by Life technologies.

### 2.16. Statistics

All data presented were means ± standard deviation (SD) of three replicates. Statistical analyses were performed by an analysis of variance (ANOVA) using SPSS software version 20. Differences between treatments were separated by the least significant difference (LSD) test at a 0.05 probability level.

## 3. Results and Discussion

### 3.1. Characterization and Identification of the Plant Specimen

**Figure 1:**
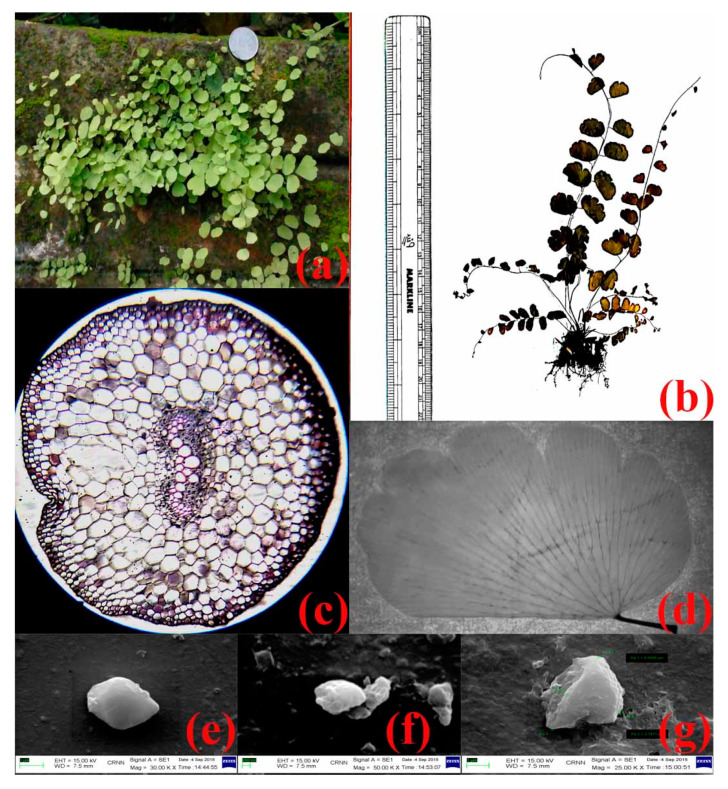
(**a**) Digital photograph of the sporophyte of *Adiantum lunulatum* Burm. f., (**b**) Photograph of the Herbarium Specimen of *A. lunulatum,* (**c**) transverse section of the stem of *A. lunulatum*, (**d**) venation pattern of the leaf of *A. lunulatum*, (**e**–**g**) scanning electron microscopic images of the spores of *A. lunulatum*.

The plant is sub-erect to erect and rhizomatous in nature. Rachis and the body seem to be glabrous and lustrous. Entire loftiness of the plant ranges between 9–18 inches and are nonarticulated (Figure 1a). Lamina is simply pinnate, lanceolate. The stem when mature appears as brownish to dark in colour. Pinnae is finely rubbery which is deep green or pale in colour, glabrous on both sides, and counts up to 10 pairs, alternate, stalked, fan-shape [62,63] (Figure 1b). Pattern of the pinnae venation is dichotomous [63] (Figure 1d). Sporophylls are not grouped in strobili, whereas sporangia are enclosed in sporocarps. Sori are not dorsal and have false indusium, sporangia formed in definite groups [64]. Transverse section of the stem reveals 3–4 layered sclerenchymatous ground tissue followed by parenchymatous cells. Xylems are of exarch and diarch type and it also appears to be V-shaped with two arms that are turned inwards [63,65,66] (Figure 1c). The SEM images of outer wall of spore appear to be rugulate which confirmed the species [67] (Figure 1e–g). Hence, conclusion made from the above recommendations identifies the specimen to be *Adiantum lunulatum* Burm. f. (*A. philippense* Linn.) of the family *Pteridaceae* [68].

### 3.2. Production and Characterization of Copper Oxide Nanoparticles

**Figure 2:**
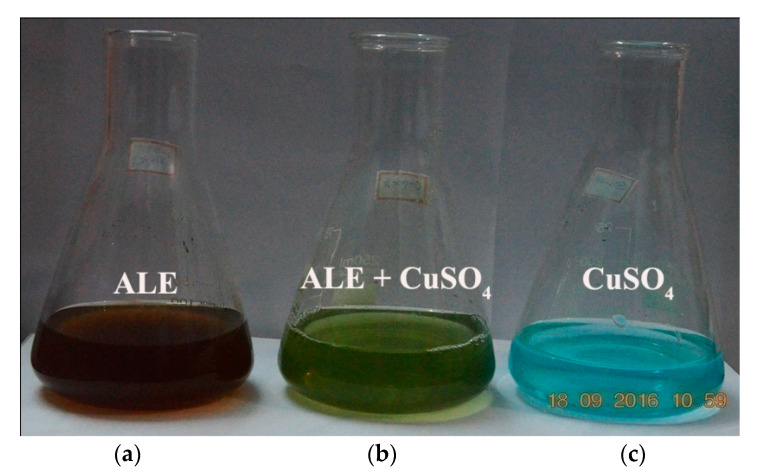
Three conical flasks containing (**a**) only the *A. lunulatum* extract (ALE), (**b**) reaction mixture of ALE and CuSO_4_ solution and (**c**) only CuSO_4_ solution, respectively.

The plant extract mediated synthesis of copper oxide nanoparticles was validated by visually monitoring three flasks containing the copper sulfate solution, plant extract of *A. lunulatum*, and the reaction mixture of the plant extract with copper sulfate solution, respectively. An instantaneous change in the colour of the reaction mixture from brown to green indicated the formation of copper-containing nanoparticles [69], whereas the plant extract and the copper sulfate solution were observed to retain their original colour (Figure 2). The colour did not change further with cumulative incubation time. The appearance of intense green colour designated the incidence of the reaction and the development of the copper oxide nanoparticles [69].

### 3.3. UV–Visible Spectroscopic Analysis Copper Oxide Nanoparticles

**Figure 3:**
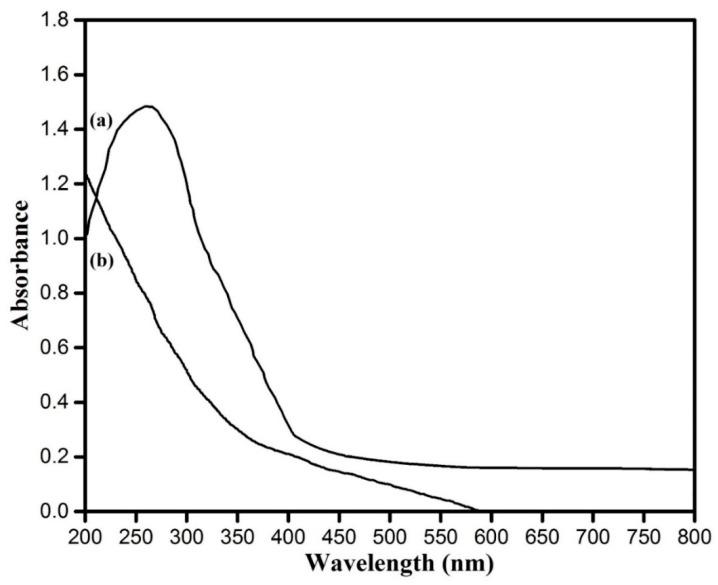
(a) UV–Visible spectra recorded as a function of reaction time of an aqueous solution of 1 mM CuSO_4_ with the *A. lunulatum* extract (ALE) and (b) only *A. lunulatum* extract (ALE).

The characteristic green colour of the reaction solution was due to the excitation of the surface plasmon vibration of copper oxide particles and provided a convenient spectroscopic signature of their formation. Both the control set showed no significant color change in the same experimental conditions. The reduction of copper sulfate was subjected to spectral analysis by using the UV–Visible spectrophotometer. This showed an absorbance peak at around 270 nm (Figure 3a), which was specific for copper oxide nanoparticles [7]. The optical density at around 270 nm was 1.5 for copper oxide nanoparticles. Simultaneously, the UV–Vis spectra of plant extract (Figure 3b) did not show such type of excitation in the said region mentioned for the copper oxide nanoparticles.

### 3.4. Particle Size Measurement of Copper Oxide Nanoparticles

**Figure 4:**
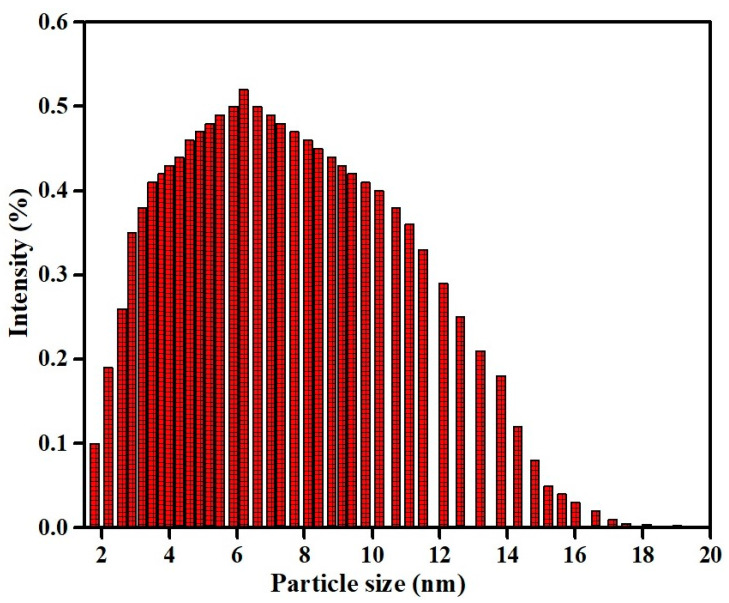
Histogram of particle size distribution as obtained from light scattering of the copper oxide nanoparticles produced by *A. lunulatum* extract (ALE).

To find out the particle size of the nanoparticles the dynamic light scattering measurement was performed. Laser diffraction had shown that particle size was found in the range of 1.5–20 nm (Figure 4). The average diameter of these copper oxide nanoparticles was calculated to be 6.5 ± 1.5 nm [70].

### 3.5. Zeta Potential of Copper Oxide Nanoparticles

**Figure 5:**
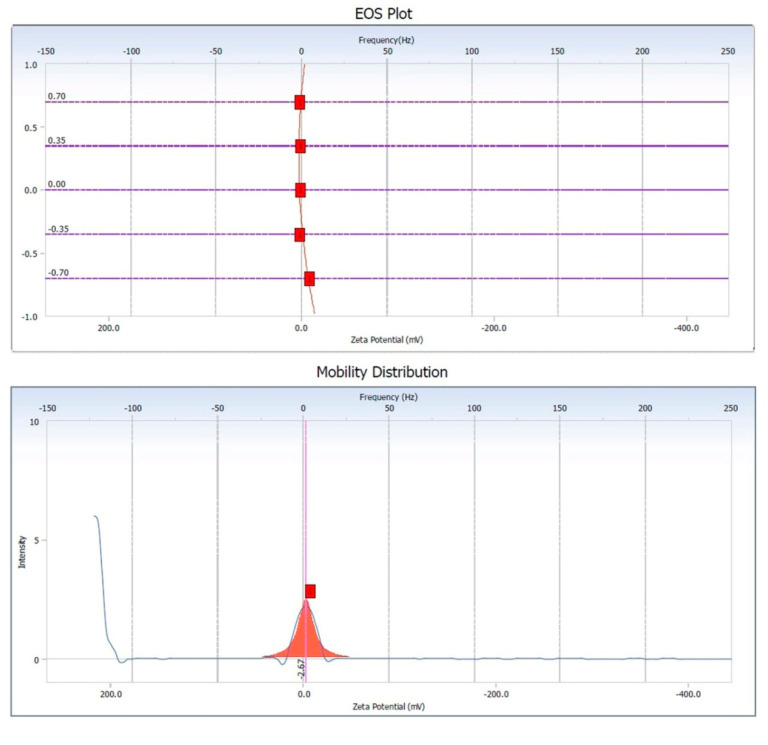
Zeta potential measurement of the copper oxide nanoparticles.

As shown in Figure 5, the zeta potential obtained from the copper oxide nanoparticles showed a negative surface charge with a value of −2.67 mV [71]. If all the particles in suspension have a negative or positive zeta potential, then they will tend to repel each other and there is little tendency for the particles to come together. The slightly negative charge on the nanoparticles was probably resulting in the high stability of the copper oxide nanoparticles without forming any aggregates when kept for an extended period of time for more than a month [6,72].

### 3.6. EDX Observation of Copper Oxide Nanoparticles

**Figure 6:**
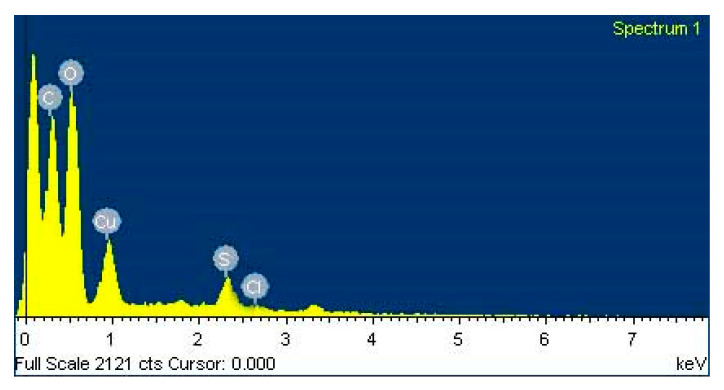
Energy dispersive spectrum of copper oxide nanoparticles.

Figure 6 shows the EDX spectrum recorded in the spot-profile mode from one of the densely-populated copper oxide nanoparticles area. In EDX spectra of copper oxide nanoparticles, two separate strong signals were observed in between 0–1 keV spectral region. The peak around 0.9 keV associated with the binding energies of copper and the peak around 0.5 keV signified with the binding energies of oxygen [73]. Therefore, the EDX spectra for the copper oxide nanoparticles confirmed the presence of copper and oxygen in the nanoparticles without any impurity of peaks [73]. However, there was other EDX peak for C and S, suggesting that they were mixed precipitates from the culture filtrate [74]. The EDX spectrum also indicates the presence of chlorine (2.6 keV) for the reason that it is usually present in nanomaterials synthesized using plant extracts [75].

### 3.7. XRD Study of Copper Oxide Nanoparticles

**Figure 7:**
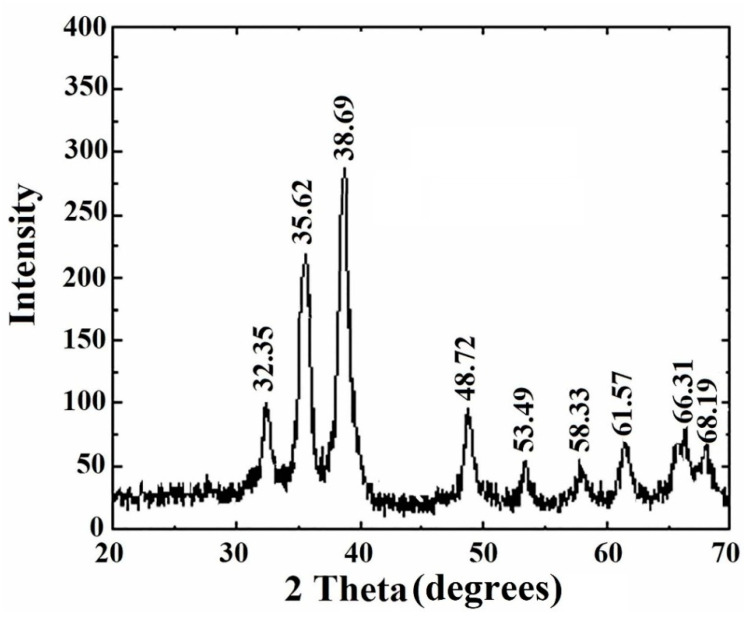
XRD spectrum of copper oxide nanoparticles.

XRD measurement often proves to be a useful analytical gizmo for the identification of the crystalline nature of the newly formed compounds and their respective phases. In this present work, the XRD diffraction patterns were detected to be at 2θ = 32.35, 35.62, 38.69, 48.72, 53.49, 58.33, 61.57, and 66.31 were assigned to (110), (111), (200), (−202), (020), (202), (−113), and (022) reflection lines, respectively of monoclinic CuO nanoparticles (JCPDS-05-0661). Thus, the XRD spectrum evidently recommended the crystalline nature of the CuONP synthesized from the plant extract of *A. lunulatum* (Figure 7) [42,76]. In this measurement, a series of diffraction peaks were observed which agreed to the Bragg’s reflection pattern of copper nanocrystals. The unambiguous background noise was undoubtedly due to the shell of protein around the nanoparticles [77].

### 3.8. FTIR Analysis of Copper Oxide Nanoparticles

**Figure 8:**
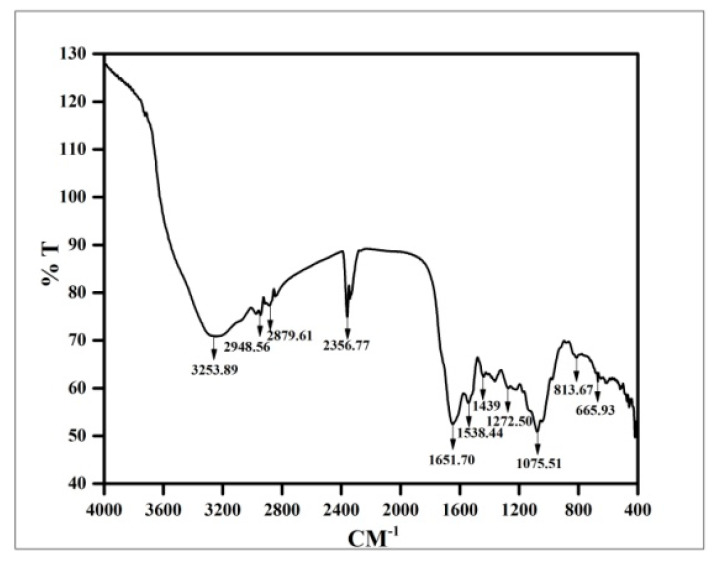
FTIR absorption spectra of copper oxide nanoparticles.

FTIR absorption spectra of biosynthesized vacuum-dried copper oxide nanoparticles are shown in the Figure 8. The spectra found at around the region of 3253 and 2948 cm^−1^ presented the bonds dueto the type movements of stretching vibrations in primary and secondary amines, respectively [71]. The broad and strong absorption band at 2356 cm^−1^ corresponded to the C–H stretching aldehydes [73]. The peaks at 1439, 1538,and 1651 cm^−1^ indicated the C–C groups derived from aromatic rings, phenols, and the conjugated carbonyl (–C=O) group stretching vibration, respectively which might come from the pteridophyte cell to the plant extract [69]. The shift of the peak near the 1600 cm^−1^ spectrum was attributed to the binding of a C=O group with the nanoparticles [78]. Simultaneously, the FTIR peaks in between 1240–1280 cm^−1^ indicated amide III band of the random coil of protein [79]. A band at 1076 cm^−1^, which corresponds to bending vibration movements in amides II, was earlier reported during the synthesis of CuS nanoparticles [80]. Three IR absorption peaks revealed the vibrational modes of CuO nanostructures in the range 700–400 cm^−1^ [81]. The foremost peaks were detected to be 525, 580, and 675 cm^−1^, respectively. The peak at 525 cm^−1^ should be a stretching of Cu–O, which matches up to the B_2u_ mode [70,81]. The peaks at 525 and 580 cm^−1^ indicated the formation of the CuO nanostructure [6]. Absorption peaks in between 900–700 cm^−1^ were also assigned to the aromatic bending vibration of C–H group [70].

From this result, it could be stated that the soluble elements present in *A. lunulatum* extract (ALE) could have acted as capping agents preventing the aggregation of nanoparticles in solution, and thus playing a relevant role in their extracellular synthesis and shaping of the quasi-spherical copper oxide nanoparticles [40,42,46,76,82,83]. In addition to that *A. lunulatum* extract (ALE) could generate different extracellular nanoparticles by a process involving the enzyme [84].

### 3.9. Transmission Electron Microscopy of Copper Oxide Nanoparticles

**Figure 9:**
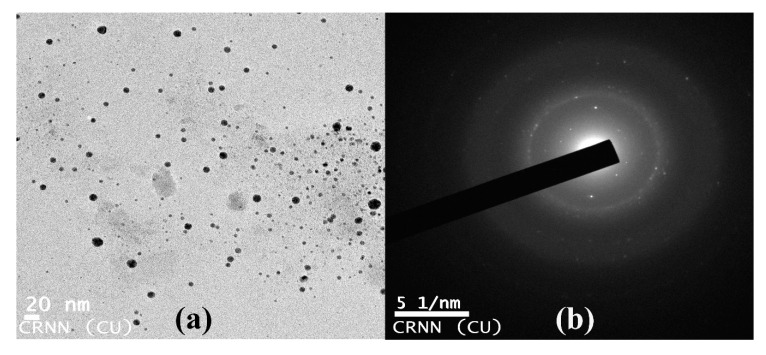
(**a**) TEM image of copper oxide nanoparticles (CuONPs). (**b**) Selected area electron diffraction (SAED) patterns of crystalline CuONPs.

The TEM image, shown in Figure 9, recorded different sizes of copper oxide nanoparticles which arose from the bio-reduction of copper sulfate solution by *A. lunulatum* extract (ALE) at room temperature (30 °C). Particles were found to be quasi-spherical, as well as mono-disperse in nature [40,71]. The measured diameter of these copper oxide nanoparticles was in the range of 1 to 20 nm [40,71]. The average diameter of these copper oxide nanoparticles was calculated to be 6.5 ± 1.5 nm. The SAED pattern showed bright circular spots which further confirmed the single crystalline property of the CuONP (Figure 9b). It was interesting to note that most of the CuONP in the TEM images were not in physical contact but were separated by a properly undeviating inter-particle distance. Due to the evolving course of the sample preparation, the detected diameter of the CuONP during TEM analysis was quite unlike from that of the results obtained from DLS measurement because CuONP were in dry state in TEM whereas in the hydrated state in DLS experiment [83,84].

### 3.10. Effects of CuONPs on Seed Germination and Growth

Significant changes were observed in seed germination percentage, root length, and seedling vigor index (Table 1). The percentage of seed germination increased in up to 1.16 and 1.13-fold in 0.01 and 0.025 mgmL^−1^ CuONPs treated seeds than control. However, it decreased slightly in 0.05 mgmL^−1^ CuONPs indicating its toxic nature. According to Nair et al. [39] seed germination percentage of *Vigna radiata* becomes significantly reduced due to toxic effects of CuONPs which was observed in our case also.

**Figure 10:**
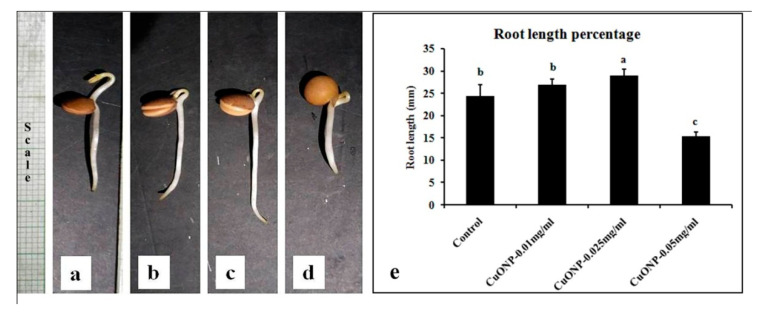
Representative samples after 72 h germination in water: (**a**) Control; (**b**) CuONP-0.01; (**c**) CuONP-0.025, and (**d**) CuONP-0.05 mgmL^−1^. (**e**) Graphical representation of root length percentage of 50 seeds in triplicate. Different letters above the bar indicate significant difference (*p* < 0.05), using Duncan’s multiple range test. Same letter above the bar denotes no significant difference between the groups.

Furthermore, root length was increased in CuONPs (0.025 mgmL^−1^) treated seedlings compared to control (Table 1). Retardation of primary root growth (Figure 10) was observed upon exposure to higher concentrations of CuONPs (0.05 mgmL^−1^) as it was observed by Nair et al. [39].

A similar trend was observed in case of vigor index of seedlings. Significant increase was found in the seeds treated with 0.01 and 0.025 mgmL^−1^ CuONPs (Table 1). However, significant reduction of vigor was observed in the seeds treated with higher concentration of CuONPs (0.05 mgmL^−1^) compared to control, indicating its toxic effects to the plants. Nair and Chung [85], also reported that the higher concentration of copper oxide nanoparticles reduced the shoot growth, weight, and total chlorophyll content in soybean.

Relative water content (RWC) of the treated roots remains close to control plants (Table 1).

### 3.11. Effects of CuONPs on Defence Related and Antioxidative Enzymes

In the present study, the expression of different defence related, as well as the antioxidative enzymes were demonstrated. All the activity of enzymes was studied and compared with the control set for inspection of the efficiency of CuONPs in the model plant system.

PO and PPO play a crucial role in triggering the hypersensitive reaction regarding cross-linking and lignifications of the cell wall and in transducing signals to adjacent unaffected cells [86,87,88,89]. On the other hand, PAL is the prime enzyme of the phenyl-propanoid pathway which initiates the biosynthesis of phenolics, phytoalexins, and lignins [90,91]. Therefore, the increased activity of PAL may contribute to the reduced percentage of disease incidence. β-1,3 glucanase is a type of PR-protein encoded by the host, that has a direct role against the fungal cell wall compounds including glucans [88].

CuONPs at a concentration of 0.025 mgmL^−1^ showed higher inductive ability of all the defence enzymes tested. However, at higher concentration (0.05 mgmL^−1^), enzyme production becomes significantly lower. Nair and Chung [85], reported that the copper oxide nanoparticles increased the peroxidase activity and lignin contents in soybean. The copper oxide exposure enhanced the lignification of root cells, which leads to the changes in root developmental process in soybean seedlings [85].

**Figure 11:**
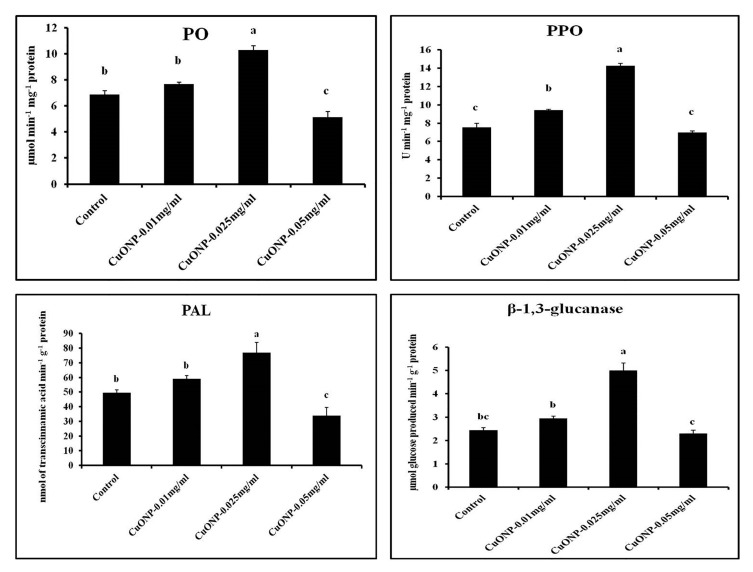
Effect of CuONPs on the production of defence enzymes in treated roots. Values represent mean ± SE of three separate experiments, each in triplicate. Sharing the same letter are not significantly different (*p* < 0.05) using Duncan’s multiple range test.

PO, PPO, PAL, and β-1,3 glucanase activity was found 1.49, 1.88, 1.56, and 2.04-fold higher in the roots treated with CuONPs (0.05 mgmL^−1^), respectively (Figure 11). A similar kind of increase in the defence related enzymes were observed by Chandra et al. [34] where tea leaves were treated with chitosan nanoparticles [34]. Higher production of defence enzymes activity may confer higher disease resistance to plants.

**Figure 12:**
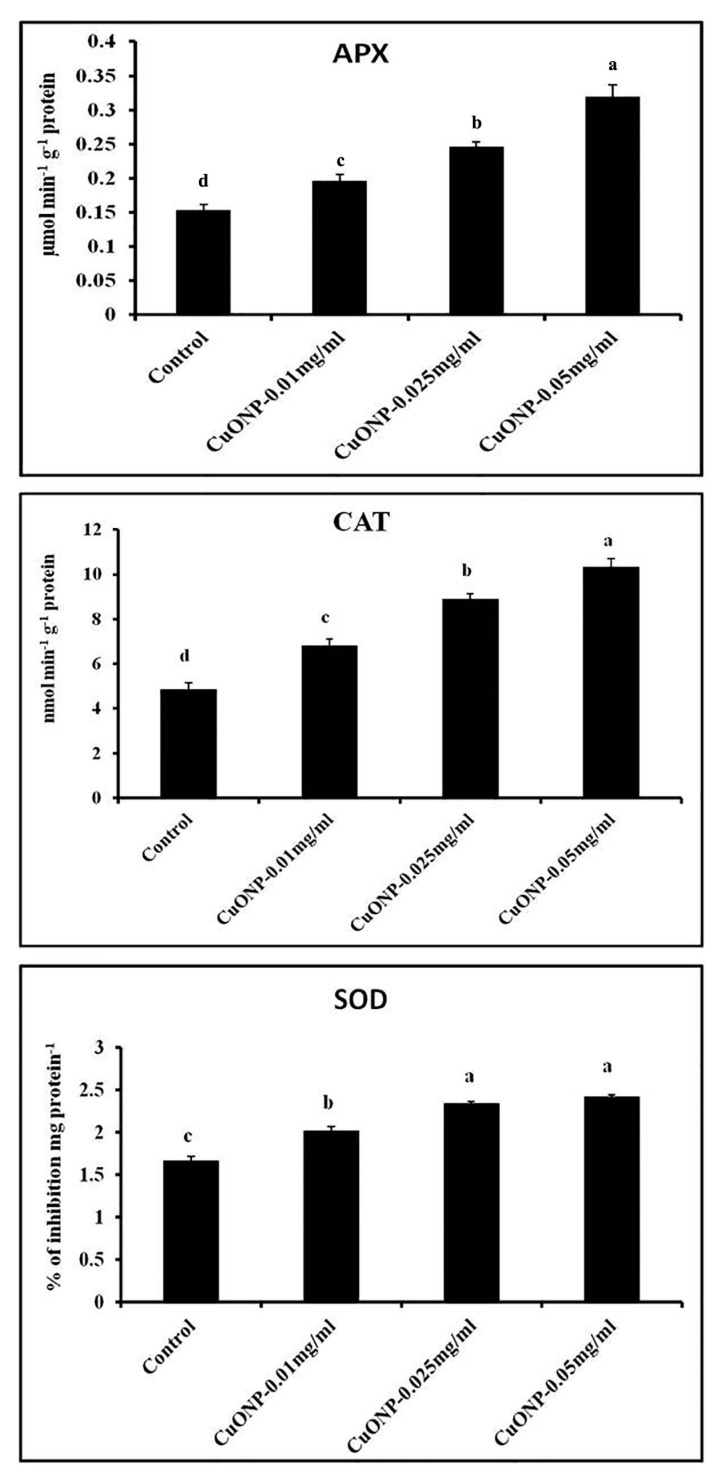
Effect of CuONPs on the production of antioxidative enzymes in treated roots. Values represent mean ± SE of three separate experiments, each in triplicate. Sharing the same letter are not significantly different (*p* < 0.05) using Duncan’s multiple range test.

CAT, APX, and SOD are the most essential components of the antioxidant system, which play a key role in removal of H_2_O_2_ from the sub-cellular compartments of plants [92]. Our results demonstrated that CAT, APX, and SOD activities have also steadily increased according to the increasing concentration of CuONPs (Figure 12). In some previous works, APX and CAT activity was found to be significantly increased in 1.0 and 1.5 mM CuONPs treatments [93,94]. The highest increase of APX, CAT, and SOD was observed in the roots treated with CuONPs (0.05 mgmL^−1^) as 2.09, 2.13 and 1.46-fold higher than control, respectively. The higher amount of antioxidative enzymes indicate a higher degree of resistance from oxidative stress. Furthermore, roots treated with CuONPs (0.025 mgmL^−1^) showed moderate level of increase for all the antioxidative enzymes.

### 3.12. Effects of CuONPs on Phenol and Flavonoid Production

**Figure 13:**
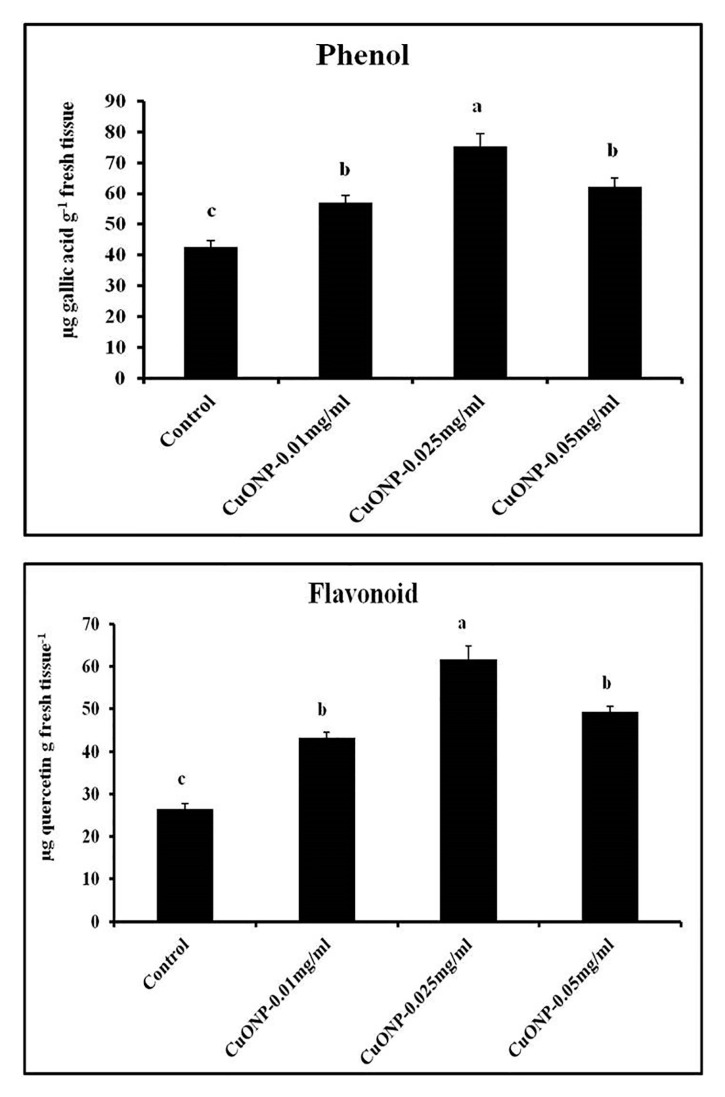
Effect of CuONPs on the production of total phenol and flavonoid in treated roots. Values represent mean ± SE of three separate experiments, each in triplicate. Sharing the same letter are not significantly different (*p* < 0.05) using Duncan’s multiple range test.

Phenols are involved in disease resistance in many ways suchas lignification of cell walls, hypersensitive cell death, etc. [95]. So, an increase of phenolic contents may give greater protection against impending pathogens. In our study, optimum concentration of CuONPs was found 0.025 mgmL^−1^, which shows higher production of phenol and flavonoid. A similar kind of observation was found in the case of tea plants when elicited with chitosan nanoparticles [34]. However, a higher concentration was found to be toxic as the production of phenolic substances become significantly lower than control (Figure 13). The highest, 1.76 and 2.33-fold increase of total phenol and flavonoid was observed in the roots treated with CuONPs (0.025 mgmL^−1^).

### 3.13. Effects of CuONPs on Lipid Peroxidation Rate and Proline Content

**Figure 14:**
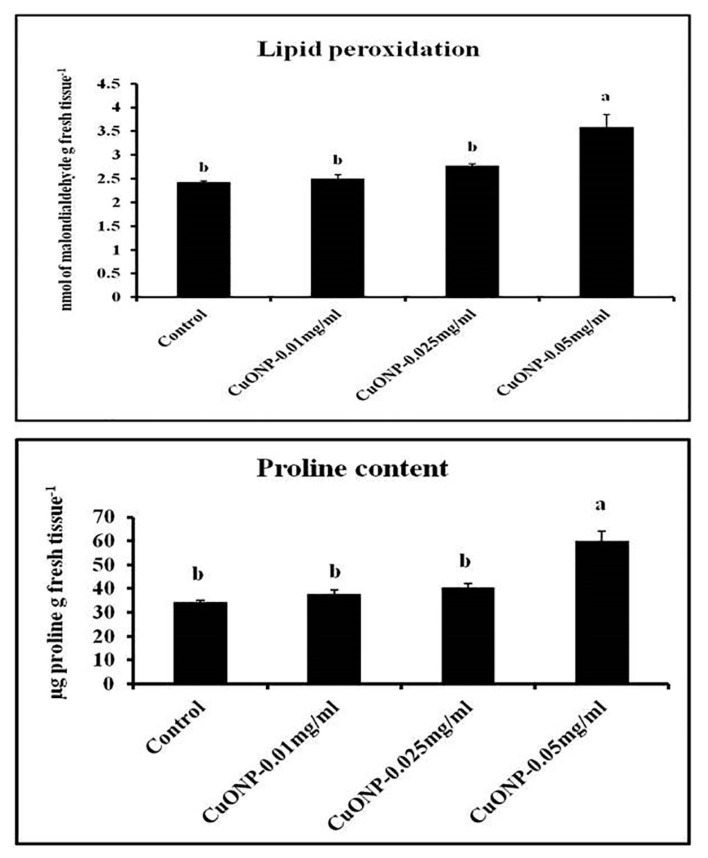
Effect of CuONPs on lipid peroxidation and proline content in treated roots. Values represent mean ± SE of three separate experiments, each in triplicate. Sharing the same letter are not significantly different (*p* < 0.05) using Duncan’s multiple range test.

Assessment of malondialdehyde (MDA) and the oxidation product of polyunsaturated fatty acid, is widely used to determine the level of lipid peroxidation [96]. An increase in proline in the plant tissue occurs in response to several abiotic stresses including salinity, drought, and frost, as well as biotic stresses [97,98]. In our case we have studied both the parameters to know whether the application of CuONPs on the roots of *Lens* generates stress or not. From the results it was evident that the higher dose of CuONPs may cause stress generation to the roots as both the parameters become significantly higher in the roots treated with CuONPs (0.05 mgmL^−1^). However, in other cases both the parameters remain as low as control. CuONPs (0.05 mgmL^−1^) showed 1.49 and 1.74-fold higher lipid peroxidation and proline content than control (Figure 14). In *Vigna radiata,* similar kinds of responses were observed by Nair et al. [39].

### 3.14. Effects of CuONPs on Nitric Oxide (NO) Production

**Figure 15:**
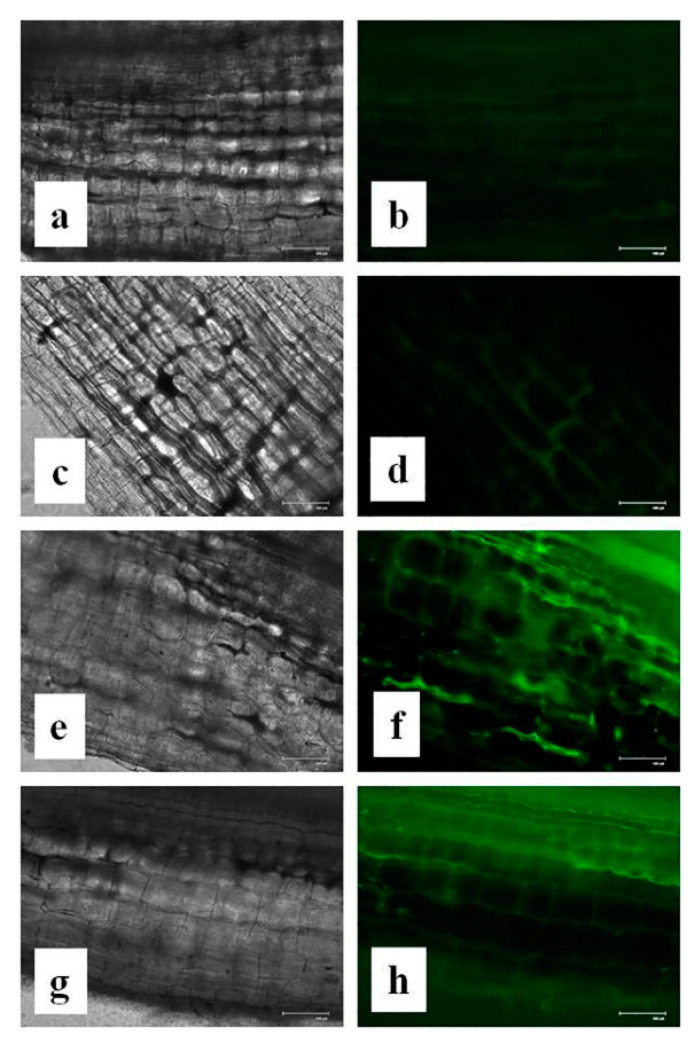
Effect of CuONPs on NO production in treated roots. (**a**,**b**) Control; (**c**,**d**) CuONPs-0.01; (**e**,**f**) CuONPs-0.025; (**g**,**h**) CuONPs-0.05 mgmL^−1^. Left column (black and white image) and right column (no generation was detected by green fluorescence).

Furthermore, a gaseous signaling molecule nitric oxide (NO) besides many of its valuable functions in plants appears to be activated after elicitor perception [99,100,101,102]. It is also believed that NO is positively involved in regulating plant defence cascades in response to various types of elicitors [103,104,105,106]. In this connection, we had tested for NO generation in our system. It was interesting to note that the production of NO in the treated roots were significantly higher than control (Figure 15). Furthermore, the higher NO production, as observed by higher green fluorescence in the root tissue, was found in CuONPs (0.025 mgmL^−1^) treated roots and was also positively correlated with higher production of defence molecules. Though, CuONPs (0.05 mgmL^−1^) showed greater fluorescence that was not correlated with higher induction of defence enzymes.

### 3.15. Effects of CuONPs on Reactive Oxygen Species (ROS) and H_2_O_2_ Production

**Figure 16:**
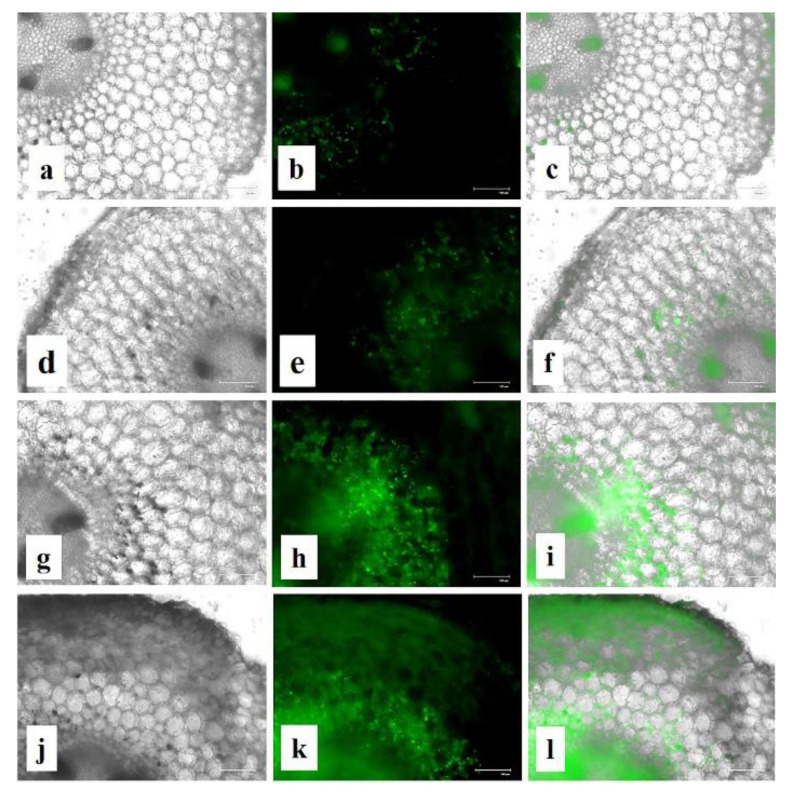
Effect of CuNPs on ROS production in treated roots. (**a**–**c**) Control; (**d**–**f**) CuONPs-0.01; (**g**–**i**) CuONPs-0.025; (**j**–**l**) CuONPs-0.05 mg mL^−1^. Left column (black and white image), middle column (green fluorescence showing ROS production) and right column (merging of white field and fluorescence figure to point out the location of ROS production in the root cells).

**Figure 17:**
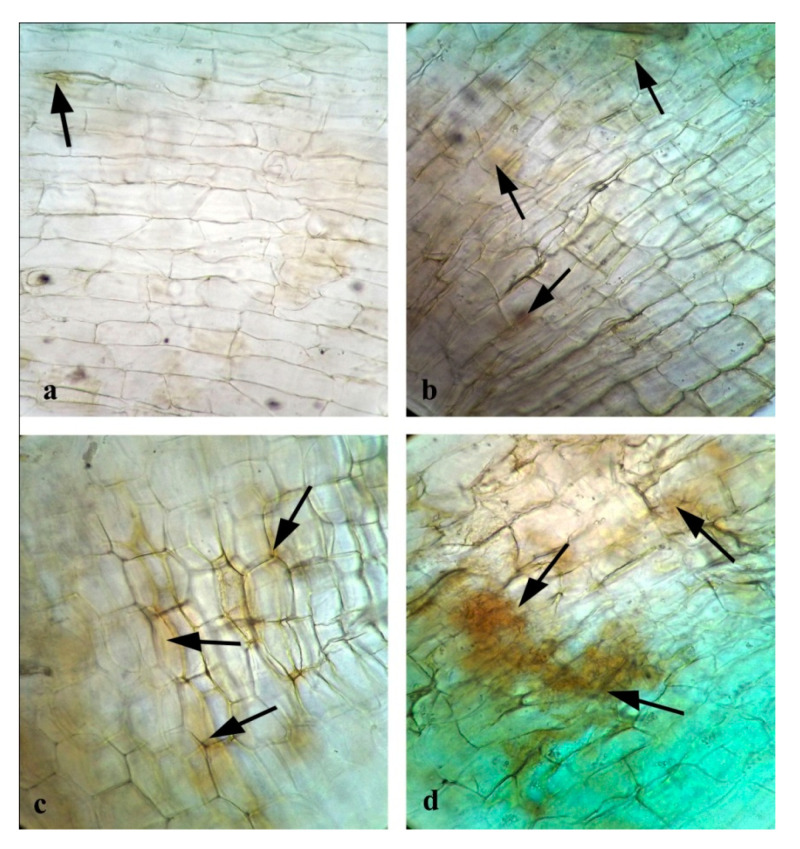
Effect of CuONPs on H_2_O_2_ production in treated roots: (**a**) Control; (**b**) CuONPs-0.01; (**c**) CuONPs-0.025; (**d**) CuONPs-0.05 mgmL^−1^.

Though past reports suggest that assembly of hydrogen peroxide (H_2_O_2_) from the oxidative burst was a prerequisite for defence gene activation [107], increased accumulation of H_2_O_2_ becomes injurious to the cells, resulting in lipid peroxidation and membrane injury [104]. To investigate whether the application of CuONPs generate oxidative stress we had also examined the ROS and H_2_O_2_ production in treated roots (Figure 16 and Figure 17). As expected, increasing concentrations of CuONPs (0.05 mgmL^−1^) showed a higher amount of ROS and H_2_O_2_ generation in treated roots, indication stress generation. However, CuONPs at a concentration of 0.01 and 0.025 mgmL^−1^, showed a moderate level of ROS and H_2_O_2_ production.

## 4. Conclusions

Green synthesis of CuONPs by using pteridophyte extract is one of the new approaches in the field of nanotechnology. From the extensive characterization it can be stated that the pteridophyte *Adiantum lunulatum* is very much efficient to produce mono-disperse CuONPs through a nontoxic way. Overall experimental results depicted that the growth of the plant is significantly altered by application of CuONPs. At the moderate concentration (0.025 mgmL^−1^), the root length was found to be increased but at higher concentration there was decrease in the length that may be due to excess CuONPs. The defence enzyme activity also becomes influenced by the application of CuONPs. At the moderate concentration (0.025 mgmL^−1^), defence enzymes such as PO, PPO, PAL, and β-1,3 glucanase activity become increased. It indicates that CuONPs at an optimum concentration has the potentiality to trigger innate immunity of plants. However, at higher concentration it becomes toxic to the plants as all the enzyme production becomes downregulated. Phenol and flavonoid levels were also induced by the application of CuONPs (0.025 mgmL^−1^). Furthermore, antioxidative enzymes such as CAT, APX, and SOD were also induced by the application of CuONPs. The higher amount of antioxidative enzymes production indicates a higher degree of resistance from oxidative stress. On the other hand, the rate of membrane lipid peroxidation and proline content remain increased with the increasing concentration of CuONPs application. However, at optimum dose (0.025 mgmL^−1^), all those parameters become close to the basal level. These results indicate lower stress generation in plants. The higher amount of ROS and H_2_O_2_ production was found in the roots treated with CuONPs (0.05 mgmL^−1^), which further validate the previous results. Interestingly, production of NO was increased up to a certain level by the application of optimum dose of CuONPs (0.025 mgmL^−1^) than control. In this connection, the higher amount of NO might influences the defence enzyme production and also checks ROS production in the treated plants. However, to establish NO as a potent mediator in this process needs further investigation.

Taken together, it can be concluded that at the optimum concentration (0.025 mgmL^−1^) innate immunity and plant vigor was induced. However, the higher concentration (0.05 mgmL^−1^) retards all the parameters instead of stress markers, ROS generation, and H_2_O_2_ production. Overall, observation suggests that, CuONPs at an optimum concentration not only have the potentiality to affect the physiological condition but also it can modulate the innate immune system of model plants such as *Lens*.

## Figures and Tables

**Table 1:** Effect of CuONPson seed germination, seedling vigor index, and relative water content in *Lens culinaris*. Values represent mean ± SD of three separate experiments, each in triplicate.

Sets	% Seed Germination	Seedling Vigor Index	RWC (%)
Control	80.77 ± 1.82 ^b^	3661.04 ± 46.41 ^c^	87.39 ± 4.37 ^a^
CuONP-0.01 mgmL^−1^	93.96 ± 1.75 ^a^	4269.4 ± 71.26 ^a^	88.38 ± 3.81 ^a^
CuONP-0.025 mgmL^−1^	91.26 ± 1.31 ^a^	4168.43 ± 48.15 ^b^	84.37 ± 5.33 ^b^
CuONP-0.05 mgmL^−1^	75.98 ± 2.41 ^c^	2541.36 ± 61.31 ^d^	81.28 ± 3.71 ^c^

Different letters within the row indicate significant difference (*p* < 0.05) from the control set using Duncan’s multiple range test. Same letter within the row denotes no significant difference between the groups.
