# Correction: Sarkar et al. Green Synthesized Copper Oxide Nanoparticles Ameliorate Defence and Antioxidant Enzymes in *Lens culinaris*. *Nanomaterials* 2020, *10*, 312

**DOI:** 10.3390/nano15231764

**Published:** 2025-11-25

**Authors:** Joy Sarkar, Nilanjan Chakraborty, Arindam Chatterjee, Avisek Bhattacharjee, Disha Dasgupta, Krishnendu Acharya

**Affiliations:** 1 Department of Botany, Dinabandhu Andrews College, Garia, Kolkata 700084, India; jsarkar80@gmail.com; 2 Department of Botany, Scottish Church College, Kolkata 700006, India; nilanjanchak85@gmail.com (N.C.); avisek0007@gmail.com (A.B.); dishadasgupta5@gmail.com (D.D.); 3 Department of Botany, University of Kalyani, Kalyani, Nadia 741235, India; arindamchatterjee206@gmail.com; 4 Molecular and Applied Mycology and Plant Pathology Laboratory, Centre of Advanced Study, Department of Botany, University of Calcutta, Kolkata 700019, India

In the original publication [1], there was a mistake in Figure 16 as published. Subfigure 16d was wrongly incorporated. In this context, the whole set of Figure 16 has been replaced in this correction. The corrected Figure 16 appears below. The authors state that the scientific conclusions are unaffected. This correction was approved by the Academic Editor. The original publication has also been updated.

## Figures and Tables

**Figure 16:**
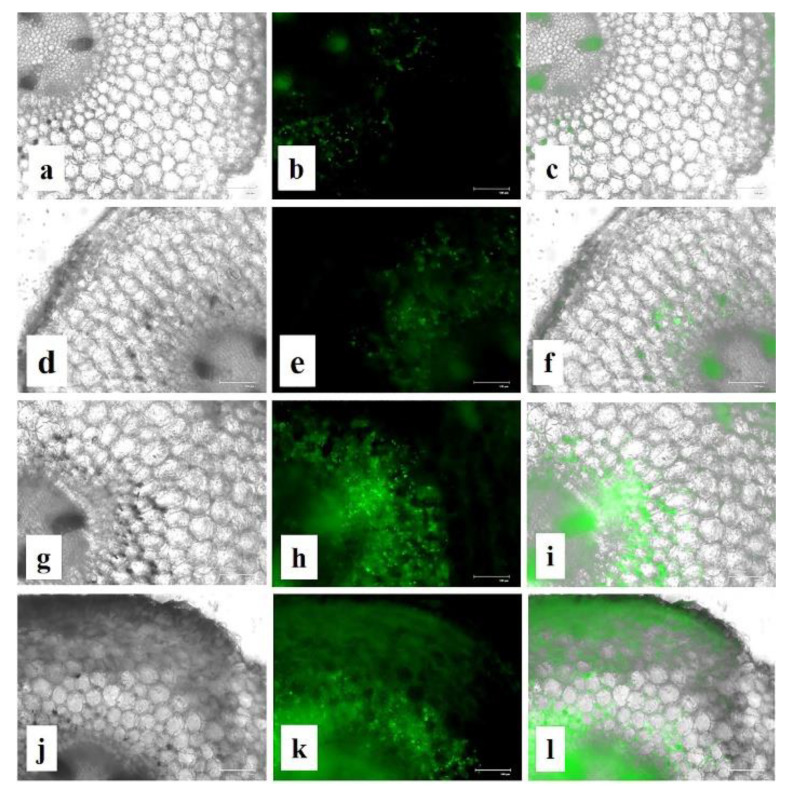
Effect of CuNPs on ROS production in treated roots. (**a**–**c**) Control; (**d**–**f**) CuONPs-0.01; (**g**–**i**) CuONPs-0.025; (**j**–**l**) CuONPs-0.05 mg mL^−1^. Left column (black and white image), middle column (green fluorescence showing ROS production) and right column (merging of white field and fluorescence figure to point out the location of ROS production in the root cells).
